# Osteoinductivity Assessment of BMP-2 Loaded Composite Chitosan-Nano-Hydroxyapatite Scaffolds in a Rat Muscle Pouch

**DOI:** 10.3390/ma4081360

**Published:** 2011-08-02

**Authors:** Benjamin T. Reves, Jessica A. Jennings, Joel D. Bumgardner, Warren O. Haggard

**Affiliations:** Biomedical Engineering Department, University of Memphis, Memphis, TN 38115, USA; E-Mails: jjnnings@memphis.edu (J.A.J.); jbmgrdnr@memphis.edu (J.D.B.); whaggrd1@memphis.edu (W.O.H.)

**Keywords:** chitosan, osteoinductivity, hydroxyapatite, BMP-2, animal study

## Abstract

The objective of this study was to evaluate the osteoinductivity of composite chitosan-nano-hydroxyapatite scaffolds in a rat muscle pouch model. Previous *in vitro* characterization demonstrated the ability of the scaffolds to promote bone regeneration and as a carrier for local delivery of BMP-2. Composite microspheres were prepared using a co-precipitation method, and scaffolds were fabricated using an acid wash to adhere beads together. To determine the *in vivo* osteoinductivity of the scaffolds, the following groups (n = 6) were implanted into muscle pouches created in the latissimus dorsi of Sprague Dawley rats: (A) lyophilized scaffolds without rhBMP-2, (B) lyophilized scaffolds with rhBMP-2, (C) non-lyophilized scaffolds with rhBMP-2, and (D) absorbable collagen sponge with rhBMP-2 (control). Groups B, C, and D were loaded with 4 mL of a 9.0 μg/mL solution of rhBMP-2 for 48 h. The rats were sacrificed after one month and samples were analyzed for amount of residual implant material, new bone, and osteoid. Although the experimental groups displayed minimal degradation after one month, all of the scaffolds contained small amounts of woven bone and considerable amounts of osteoid. Approximately thirty percent of the open space available for tissue ingrowth in the scaffolds contained new bone or osteoid in the process of mineralization. The ability of the composite scaffolds (with and without BMP-2) to promote ectopic bone growth *in vivo* was demonstrated.

## 1. Introduction

Of the approximate eight million bone fractures that occur in the United States each year, 5–10% of these fractures will result in delayed healing or non-union [1]. The current gold standard for augmenting healing in these troublesome fractures is the use of autografts. However, autografts suffer from a number of drawbacks including surgical site infection, difficulty shaping the graft to fit the defect, donor site morbidity, and limited graft material [2,3,4]. Allografts are not as effective as autografts and transmission of disease from the donor remains a concern [3,4,5]. Demineralized bone matrix displays extremely varying rates of effectiveness [6,7]. For these reasons, much current research has focused on the development of bone regeneration scaffolds that can be used as bone graft substitutes. These scaffolds are designed to provide a matrix to which osteoblasts can attach and proliferate. Ideally, the scaffolds will provide mechanical strength initially and then degrade as new bone is deposited [8,9]. Our laboratories have developed composite chitosan-nano-hydroxyapatite scaffolds to enhance fracture healing [10,11,12].

After cellulose, the most abundant biopolymer is chitin. Chitin is found in the exoskeletons of crustaceans and insects [13,14]. The deacetylated derivative of chitin is known as chitosan, and chitosan is a carbohydrate copolymer composed of glucosamine and N-acetyl-D-glucosamine units joined by β-1,4 glycosidic bonds. If the copolymer contains more than 50% glucosamine units, it is referred to as chitosan; whereas, it is still called chitin if it retains more than 50% N-acetyl-D-glucosamine monomers [15,16]. Chitosan has a number of properties including biocompatibility, biodegradability, mucoadhesiveness, and wound healing capabilities that make it useful as a biomaterial. Chitosan is very versatile and can be prepared as films, gels, sponges, beads, fibers and other forms and has been used in various applications including wound healing, drug delivery, and bone tissue engineering [14,15,16,17,18,19,20].

Ideally, scaffolds used for bone regeneration need mechanical strength [8]. The pores of the scaffold must remain open to allow tissue ingrowth into the interior of the scaffolds and to maintain good nutrient/waste exchange [21,22]. Our composite scaffolds incorporate the strength and hardness of hydroxyapatite with the toughness and flexibility of chitosan. Hydroxyapatite is the main inorganic component of bone and has been used in coatings to improve osteoblast response to implants [9,14,23,24,25,26]. In addition, our labs have demonstrated the enhanced bone regenerative capacity of composite scaffolds over chitosan-only scaffolds [10,11].

To further increase the bone regenerative properties of graft substitutes, the scaffolds can also serve as a carrier for the local delivery of growth factors [21,27,28]. Bone morphogenetic protein-2, BMP-2, has been widely investigated for augmenting fracture healing due to its pleotropic nature. BMP-2 recruits stem cells to the fracture site, promotes angiogenesis, and causes differentiation of the stem cells into osteoblasts [27,29,30,31,32,33]. We have previously shown that increased BMP-2 loading can be achieved using composite scaffolds instead of chitosan-only scaffolds. We also demonstrated that even further BMP-2 loading can be achieved using lyophilization (freeze-drying) to increase the surface area of the scaffolds [12].

The objective of this investigation was to evaluate the osteoinductivity of BMP-2 loaded composite chitosan-nano-hydroxyapatite scaffolds in a rat muscle pouch model. The murine muscle pouch model is a well-established model for determining the osteoinductivity of materials [34,35,36]. We hypothesized that the lyophilized composite scaffolds would induce the most bone formation due to enhanced BMP-2 loading.

## 2. Results and Discussion

**Figure 1:**
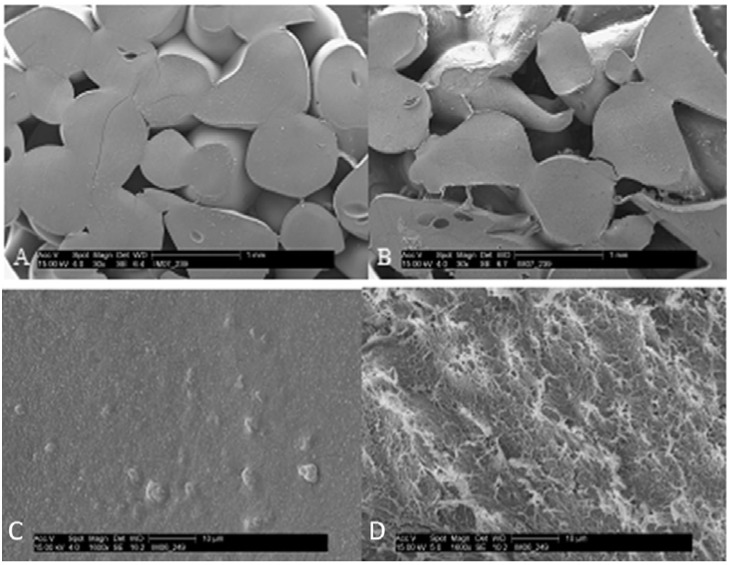
Scanning electron micrograph (SEM) images of composite scaffolds and microspheres. (**A**) Non-lyophilized composite scaffold, 30×; (**B**) Lyophilized composite scaffold, 30×; (**C**) Surface of non-lyophilized composite microsphere, 1600×; (**D**) Surface of lyophilized composite microsphere, 1600×. Note the slightly increased porosity of the lyophilized scaffolds. The surface of the lyophilized microsphere is considerably rougher than the surface of the non-lyophilized microsphere. (Reprinted with permission of John Wiley & Sons, Inc. Original figures located in Journal of Biomedical Materials Research Part B: Applied Biomaterials, Volume 90B, Issue 1, Copyright © 2009.)

Porous composite scaffolds were successfully prepared by fusing chitosan-nano-hydroxyapatite beads together using an acid wash (Figure 1). The initial porosity of the non-lyophilized and lyophilized scaffolds were 35.8 ± 2.1% and 53.6 ± 3.6%, respectively [12]. Thus, the lyophilized scaffolds are slightly more porous than the non-lyophilized scaffolds. It has been suggested that a minimum porosity of thirty percent is required for bone regeneration [37]. In addition, the 100–800 micron pore diameters of the composite scaffolds are suitable for bone regeneration [10], since pores of at least one hundred microns are required for osteogenesis [9].

Following implantation into rat muscle pouches for one month, the osteoinductive potential of the composite scaffolds was determined. Using BIOQUANT OSTEO II imaging software, the amount of residual implant material, osteoid, and new bone as a percent of total implant area were quantified (Table 1). The remaining space in the implant area was occupied by fibrous or muscle tissue.

**Table 1:** Composite Scaffold Performance.

	Scaffold (%)	Osteoid (%)	Bone (%)
Lyophilized (no rhBMP-2)	65.2 ± 3.7	8.8 ± 2.6	1.8 ± 0.8
Lyophilized with rhBMP-2	59.2 ± 6.1	10.4 ± 1.2	1.2 ± 0.3
Non-lyophilized with rhBMP-2	71.8 ± 3.0	7.7 ± 2.4	1.0 ± 0.7
Collagen Sponge with rhBMP-2	N/A	N/A	94.0 ± 4.4^a^

N/A: Not applicable; a: Bone and marrow.

**Figure 2:**
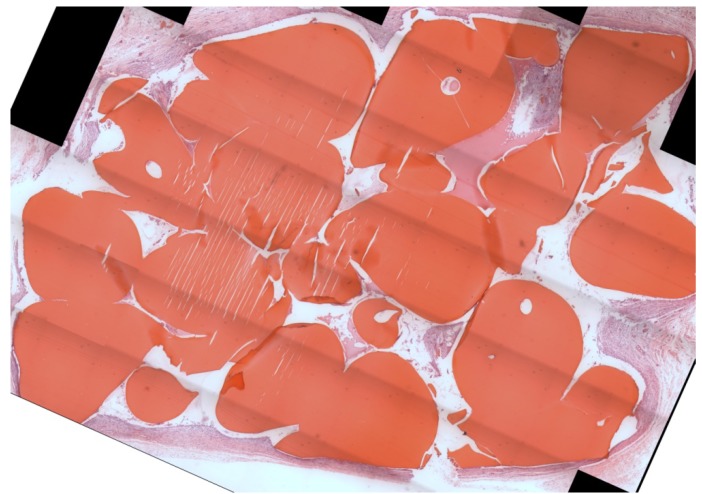
Light microscope image of representative histology section, H&E stain. The bright red objects are residual scaffold material. Note that most of the implant space is still occupied by scaffold material. Some histological artifacts are present in this particular section. Scaffold type was non-lyophilized with rhBMP-2.

As seen in Table 1 and displayed in Figure 2, the majority of the implant space was still occupied by scaffold material after one month. Recall that the initial porosities of non-lyophilized and lyophilized scaffolds were 35.8 ± 2.1% and 53.6 ± 3.6%, respectively [12]. Thus, no degradation was observed, and the scaffolds actually appeared to increase in mass slightly during this experiment. This slight increase may be due to histological artifacts. Also, the amount of remaining chitosan was determined by evaluating thin slices through the scaffold; whereas, the initial porosity was determined using slices through the entire scaffold obtained by Micro-CT. Using the same 92.3% degree of deacetylation (DDA) chitosan, Chesnutt *et al*. observed no measurable degradation during a two-week *in vitro* degradation study and very minimal degradation during a 12-week rat calvarial defect model [10,11].

As previously discussed, controlled degradation is an important characteristic of bone regenerative scaffolds. If the scaffolds do not degrade in a timely manner, extensive new bone formation will be prevented due to lack of space [21,38,39]. The 92.3% DDA chitosan used in this study was chosen due to its good mechanical properties; however, chitosan with a lower DDA has been shown to degrade considerably faster [18,40]. As DDA decreases, so does the crystallinity of the chitosan. This lower crystallinity allows lysozyme, the main enzyme responsible for chitosan degradation *in vivo*, easier access to the glycosidic bonds between the monomers [18,41]. Other potential methods for increasing scaffold degradation include using a lower molecular weight chitosan, decreasing the weight percent of chitosan, and using other solvent acids.

**Figure 3:**
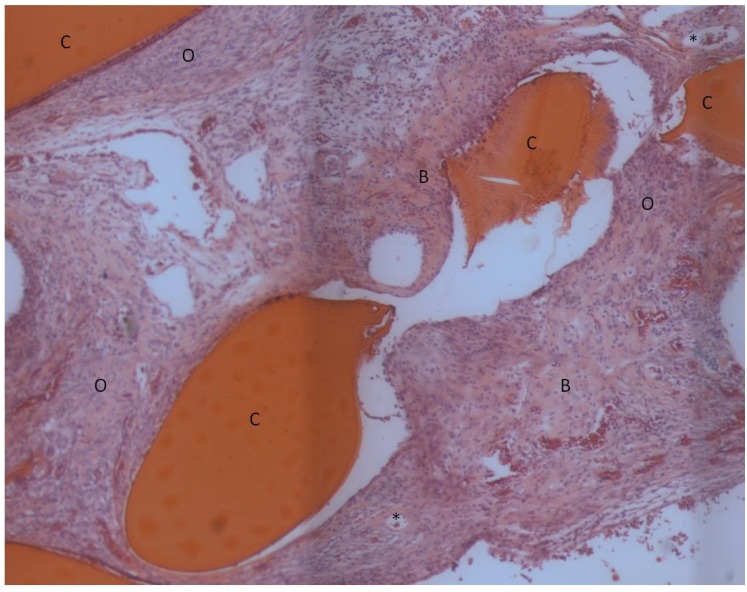
Light microscope image of lyophilized scaffold without rhBMP-2, H&E stain. The bright red objects are residual scaffold material. New bone tissue near and adjacent to the scaffold is present. An extensive amount of osteoid material which is in the process of becoming mineralized is also evident. Some capillaries with red blood cells in their interior can be seen. C: residual chitosan, B: bone, O: osteoid, *: capillaries.

**Figure 4:**
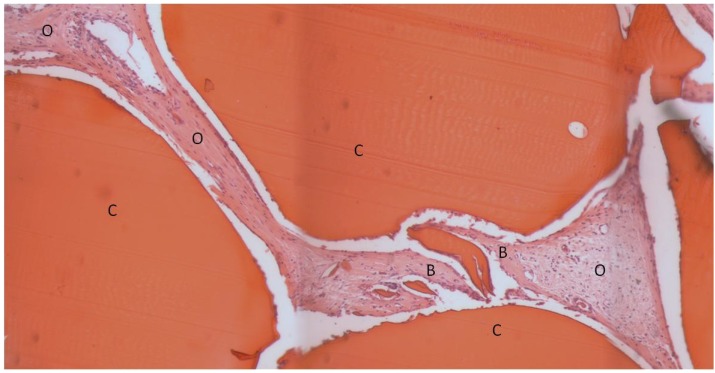
Light microscope image of lyophilized scaffold with rhBMP-2, H&E stain. The bright red objects are residual scaffold material. Some bone has formed in the pores of the scaffold. The remaining tissue appears to be osteoid material in the process of becoming mineralized. C: residual chitosan, B: bone, O: osteoid.

**Figure 5:**
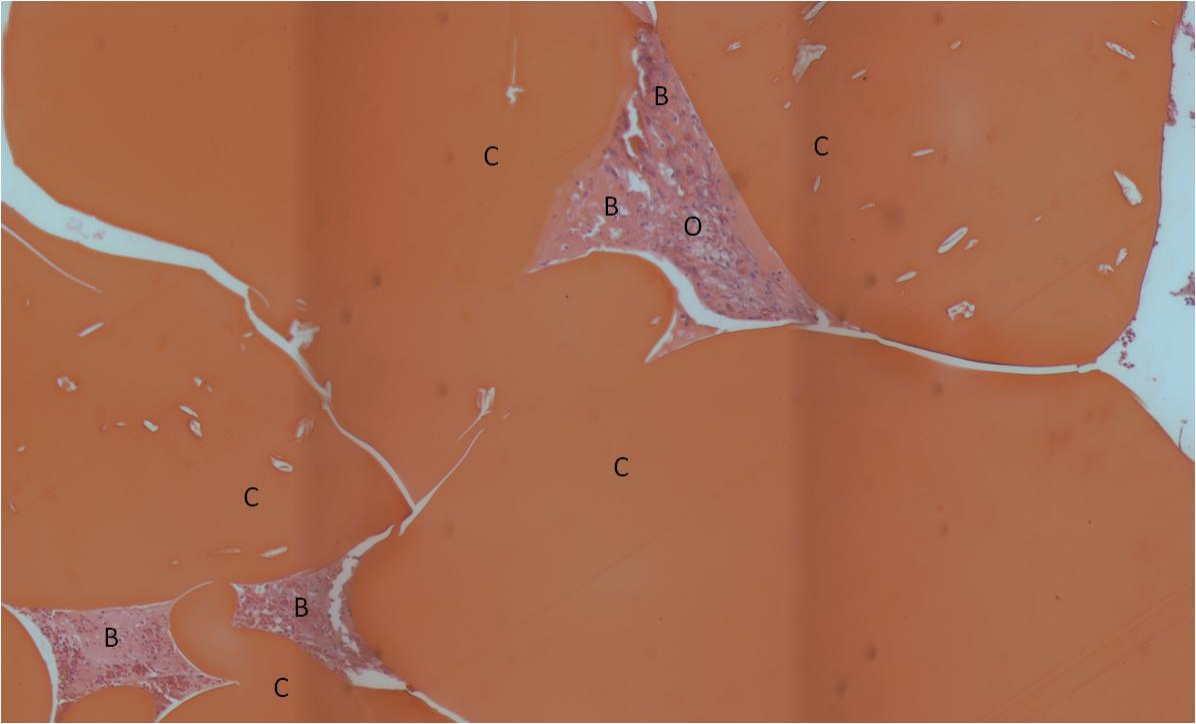
Light microscope image of non-lyophilized scaffold with rhBMP-2, H&E stain. The bright red objects are residual scaffold material. Bone and osteoid are occupying the pores of the scaffold. C: residual chitosan, B: bone, O: osteoid.

The composite chitosan-nano-hydroxyapatite scaffolds appeared to be very biocompatible. No adverse tissue reactions were observed. A small amount of new bone was seen in the implant area for all three groups (Figure 3, Figure 4, and Figure 5).

The amount of new bone was not statistically different between the groups (p = 0.16). It was somewhat surprising that new bone was found in the lyophilized scaffolds without BMP-2. Both chitosan [14,15,21] and hydroxyapatite [4,35,42] are considered to be osteoconductive, meaning that they are able to support the attachment and proliferation of bone cells but do not have the ability to cause stem cells to differentiate into osteoblasts. Furthermore, various composite chitosan-hydroxyapatite preparations have been shown to be osteoconductive [43,44,45,46]. However, some *in vitro* and *in vivo* data claiming hydroxyapatite to be osteoinductive does exist. Lin *et al*. demonstrated the ability of porous hydroxyapatite to induce expression of genes for alkaline phosphatase, osteocalcin, and Type I collagen in uncommitted pluripotent C3H10T1/2 mouse stem cells [47]. Porous nanohydroxapatite/polyamide 66 scaffolds were found to be osteoinductive in New Zealand white rabbit muscle pouches by Xu *et al*. [48]. Hydroxyapatite has also been shown to be osteoinductive in large animals. Ripamonti *et al*. have demonstrated the ability of hydroxyapatite disks derived from coral and hydroxyapatite disks prepared using a solid-state reaction to be osteoinductive when implanted intramuscularly in baboons [49,50]. A number properties including topography, surface energy, surface area, and crystallinity of hydroxyapatite and other calcium phosphate materials are crucial in determining their osteoconductive and osteoinductive potentials [25,51,52,53,54]. In this study, the scaffolds appear to have the right combination of these surface chemistry and microarchitecture properties to impart some degree of osteoinductivity. It should also be noted that in the current experimental design, each rat received two scaffolds (one in each bilateral pouch); thus, it is possible that BMP-2 was able to diffuse from one implant site to another. However, proteolytic enzymes are expected to quickly degrade any diffusing BMP-2, and the half-life of BMP-2 is 7-16 minutes [55]. Thus, we believe the presence of new bone in the scaffold group without BMP-2 is an indication that our composite scaffolds are very suitable for bone regeneration. It is also very promising that new bone was observed in direct contact with the composite scaffolds.

While the amount of new bone observed for the composite scaffolds was low, more mineralized tissue might have been observed if a later timepoint had been used. However, considerable amounts of osteoid were observed for the three experimental groups. Thus, significant regions of unmineralized matrix which were expected to be later mineralized and converted to bone were observed. Since the composite scaffolds did not degrade and occupied a large portion of the implant area, the amount of new bone and osteoid were normalized to the amount of space available for tissue formation by calculating the bone tissue index (BTI) using the following equation:

(1) $$BTI=\frac{\left(Bone+Osteoid\right)}{\left(100-Scaffold\right)}\times 100\%$$

Thus, the BTI is an indicator of how much of the open pore space in the implant area was filled in with new bone or was in the process of being converted to bone after one month. Table 2 displays the BTI for the experimental groups.

**Table 2:** Bone Tissue Index (BTI) Values for Composite Scaffolds.

	Bone Tissue Index (%)
Lyophilized (no rhBMP-2)	30.6 ± 8.5
Lyophilized with rhBMP-2	28.8 ± 5.4
Air-dried with rhBMP-2	30.5 ± 5.7

The values of the BTIs for all three groups were approximately thirty percent and were not statistically different (p = 0.90). This degree of bone and osteoid formation is comparable to that observed in studies with other non-degrading porous implant materials. Baril *et al*. observed 20–25% bone ingrowth after six weeks into the pores of titanium implants with approximately 50% porosity [56]. Only 11.4 ± 2.4 and 10.5 ± 1.8% of bone ingrowth as a percent of void space after twelve weeks was observed by Willie *et al*. in titanium foam implants with porosities of 74.4 and 79.0%, respectively [57]. Following implantation of hydroxyapatite implants with 50% porosity into the femoral condyles of rabbits, Wang *et al*. observed 2.54 ± 0.59% bone ingrowth (as a percent of total defect area) after three weeks [58]. This value is similar to the bone ingrowth observed after four weeks in our chitosan-nano-hydroxyapatite scaffolds. However, Wang *et al.* found no bone in the interior region of their implants; whereas, new bone was found in the interior region of our scaffolds directly adjacent to composite beads. After three weeks of implantation in rat tibial defects, Zreiqat *et al*. observed approximately 25% bone ingrowth into pores of ceramic Hardystonite (77.5% porosity) and Sr-Hardystonite (78% porosity) [59]. Thus, we believe that our composite chitosan-nano-hydroxyapatite scaffolds have similar or potentially improved osteogenic capacity compared to the discussed biomaterials.

The experimental group containing lyophilized scaffolds with BMP-2 was expected to perform the best in this study due to increased BMP-2 loading. However, our hypothesis was not confirmed. The lack of degradation by the scaffolds may have reduced the effectiveness of BMP-2 delivery, since there was little space for new bone to be deposited. Also, the success of BMP-2 delivery depends upon the release profile of the specific carrier [60,61,62]. Although there is some debate over what type of delivery profile is optimal for promoting osteogenesis, evidence suggests that a small to moderate burst release followed by sustained release of BMP-2 may be most effective [63,64,65]. *In vitro* characterization of BMP-2 elution from the composite scaffolds revealed a large burst release in which the majority of the growth factor was released within the first few days [12]. Perhaps more new bone would have been seen in the BMP-2 groups if a more optimal release profile occurred. We believe that the 36 μg of BMP-2 per implant used in this study was an appropriate amount. Levels as low as 1.0 μg [60] to as high as 150 μg [66] of BMP-2 have been used successfully in similar murine ectopic bone models. Engstrand *et al.* observed greatly increased bone volume when 50 μg of BMP-2 was delivered compared to 10 μg [61]. An amount of 20 μg of BMP-2 maintained ectopic bone formation better than 10, 5, or 2.5 μg in a study by Lee *et al*. [67].

Somewhat surprisingly, the increased total porosity of the lyophilized scaffolds did not result in increased new bone formation compared to non-lyophilized scaffolds. The non-lyophilized scaffolds did have the lowest value for amount of new bone (Table 1), but this value was not statistically significant. The differences in porosity between the non-lyophilized and lyophilized scaffolds (35.8 ± 2.1 and 53.6 ± 3.6, respectively) may not have been enough to considerably alter bone tissue formation. Although only a small amount of new bone was observed in this study, our composite scaffolds were able to promote and sustain ectopic bone growth. Furthermore, bone was found in the interior of the scaffolds and in direct contact with the scaffolds. We believe that our technology can be modified to produce scaffolds with a faster degradation rate and that these scaffolds will be able to support extensive bone formation.

The BMP-2 loaded absorbable collagen sponge used as a positive control in this study completely degraded after one month and the implant area was filled in with bone and adipose tissue. Bone or adipose tissue (indicative of marrow formation) filled in 94.0 ± 4.4% of the implant area. No adverse tissue response to the material was observed (Figure 4).

**Figure 6:**
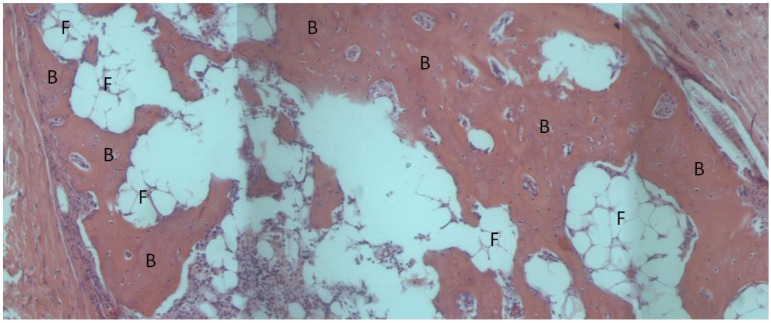
Light microscope image of absorbable collagen sponge, H&E stain. The collagen sponge has completely resorbed and is being replaced with bone. Fat globules indicative of marrow formation can also be seen. B: bone, F: fat globules.

The BMP-2 loaded absorbable sponge promoted extensive osteogenesis as expected. This sponge is used clinically to promote interbody spinal fusion and in the treatment of open tibial fractures. Although the sponge degrades quickly to allow extensive new bone formation, the collagen sponge cannot provide mechanical support to the fracture site and must be used in conjunction with hardware to prevent collapse of the defect [68].

## 3. Experimental Section

### 3.1. Composite Microsphere and Scaffold Fabrication

Composite microspheres and scaffolds were prepared as previously described [12]. Firstly, microspheres were fabricated using a co-precipitation method. A solution containing 3.57 weight percent (wt. %) chitosan (92.3% DDA; M_v_ = 4.66 × 10^5^ g/mole; Vanson, Redmond, WA), 0.1 M CaCl_2_, and 0.06 M NaH_2_PO_4_ (Ca:P ratio = 1.67) was prepared in 2 wt. % acetic acid. A precipitation solution (pH = 13) containing 20 wt. % NaOH, 30 wt. % methanol, and 50 wt. % water was prepared. Using a syringe pump, the chitosan solution was added dropwise through 21G needles into the precipitation solution, and spherical microspheres immediately formed. The composite microspheres were allowed to wash in the precipitation solution for 24 h to allow crystalline hydroxyapatite to form. The microspheres were then washed in deionized (DI) water until a neutral pH (<7.5) was achieved.

Porous composite scaffolds were formed by adhereing the microspheres together. The microspheres were briefly rinsed in 1 wt. % acetic acid and packed into 13mm-diameter plastic tubes to dry. Once the scaffolds had completely dried, they were rehydrated in DI water and cut into cylinders with an approximate height of 4mm and diameter of 5.75 mm. Some of the scaffolds were allowed to dry again; whereas, some of the rehydrated scaffolds were placed in a freezer at −20 °C and subsequently lyophilized in a 2.5 L Labconco freeze-dryer. All scaffolds were sterilized using 25 kGy gamma irradiation.

### 3.2. Scaffold Preparation for Surgery

The following groups (n = 6) were prepared for implantation into rat muscle pouches: (A) lyophilized scaffolds without rhBMP-2, (B) lyophilized scaffolds with rhBMP-2 (Genetics Institute, Cambridge, MA), (C) non-lyophilized scaffolds with rhBMP-2, and (D) absorbable collagen sponge (Medtronic, Inc., Memphis, TN) with rhBMP-2. The collagen sponge was aseptically cut into pieces 1 cm × 1 cm. A 9.0 μg/mL solution of rhBMP-2 was prepared in sterile water. An amount of 4 mL of rhBMP-2 solution was added to Groups B, C, and D for 48 h in sterile glass vials. After 48 h, the loading solution was aspirated and the scaffolds were stored in the glass vials at 4 °C until the surgeries.

### 3.3. Animal Surgeries

All procedures described were approved by the Institutional Animal Care and Use Committee at the University of Memphis (Protocol #0639) and conform to the laws and regulations of the United States. Upon arrival, twelve three-to-four month-old male Sprague Dawley rats were allowed to acclimate for one week. For surgery, rats were anesthesized with a subcutaneous injection of telazol. The back of each rat was shaved and scrubbed with betadine. A single 1.5 cm incision was made through the skin on each side of the midline. In each incision, a 1 cm pouch was created in the latissimus dorsi muscle using blunt dissection. A single randomized test specimen was implanted in each muscle pouch. Following implantation, the muscle and skin incisions were closed with 4-0 Vicryl sutures.

The rats were sacrificed after one month. The implants and surrounding tissue were excised and stored in 10% formalin. Following decalcified histological processing, three sections of each sample were stained with hematoxylin and eosin. Using the BIOQUANT OSTEO II v.8.10.20 imaging system, the total implant area in each section was identified. Sections were analyzed for amount of residual implant material, new bone, and osteoid as a percent of total implant area. One-way analysis of variance (ANOVA) was performed to determine statistical significance between groups with p < 0.05 considered significant.

## 4. Conclusions

The ability of composite chitosan-nano-hydroxyapatite scaffolds to promote ectopic bone formation in a rat muscle pouch was demonstrated. Interestingly, both BMP-2 loaded scaffolds and scaffolds without BMP-2 were also able to promote osteogenesis. Increased bone formation due to local BMP-2 delivery was not observed, possibly due to the lack of degradation exhibited by the scaffolds. Also, the large burst effect release of BMP-2 from the composite scaffolds may not have been the optimal elution profile to promote ectopic bone growth. Although new bone formation in the total implant area was minimal (less than 2%), roughly thirty percent of the void space of the composite scaffolds contained bone or osteoid after one month. An absorbable collagen sponge loaded with BMP-2 used as a positive control completely degraded after one month, and 94.0 ± 4.4% of the implant area contained new bone or marrow.
